# Wild canids as reservoirs of zoonotic* Bartonella* species in Israel

**DOI:** 10.1186/s13071-026-07576-x

**Published:** 2026-07-15

**Authors:** Jonathan Gonçalves‐Oliveira, Sharon Horesh, Anastasia Antonovskaia, Yaarit Nachum-Biala, Roi Lapid, Tomer Nissimyan, M’hammed Sarih, Maria Bourquia, Shimon Harrus

**Affiliations:** 1 Koret School of Veterinary Medicine, The Hebrew University of Jerusalem, Rehovot, Israel; 2 The Israel Nature and Parks Authority, Jerusalem, Israel; 3 Laboratory of Vector-Borne Diseases, Pasteur Institute, Casablanca, Morocco; 4 Parasitology and Parasitic Diseases Unit, Department of Animal Pathology and Public Health, Hassan II Institute of Agronomy and Veterinary Medicine, Rabat, Morocco

**Keywords:** *Bartonella*, Wild canids, Golden jackal, Zoonoses, One Health

## Abstract

**Background:**

*Bartonella* species are Gram-negative bacteria transmitted by blood-feeding arthropods and maintained in a wide range of mammalian hosts worldwide. They are emerging and re-emerging pathogens at the wildlife-domestic, animal-human interface. Wild canids are considered important reservoirs and sentinels for vector-borne pathogens, including zoonotic species of *Bartonella*. This study evaluated the eco-epidemiological traits and diversity of *Bartonella* infections among wild canids from Israel.

**Methods:**

Between 2018 and 2025, blood samples from wild canids were collected across 158 sampling sites in Israel. Three canine species were included: *Canis aureus* (golden jackal), *Canis lupus* (gray wolf), and *Vulpes vulpes* (red fox). DNA samples were screened for *Bartonella* spp. using real-time qPCR targeting the ITS locus, followed by amplification of *gltA* and *ssrA* gene fragments for ITS-positive samples. Species, sex, season, climate zone, and sampling site were recorded for each animal and evaluated as explanatory variables for *Bartonella* infection status. Associations with *Bartonella* positivity were assessed using generalized linear models (GLMs) to determine whether infection prevalence varied according to host and environmental factors.

**Results:**

A total of 473 blood samples were analyzed, including 454 golden jackals, 12 red foxes, and 7 gray wolves. Overall *Bartonella* prevalence was 24.9% (113/454) in golden jackals and 33.3% (4/12) in red foxes, while no positive wolves were detected. Four *Bartonella* species were identified: ‘*Candidatus* Bartonella merieuxii’, *Bartonella rochalimae*, *Bartonella henselae*, and *Bartonella vinsonii* subsp. *berkhoffii*. ‘*Ca*. B. merieuxii’ was the predominant species detected. Among golden jackals, prevalence remained relatively stable across seasons and was higher in Hot Summer Mediterranean (Csa) and Hot Semi-arid (BSh) climate zones, whereas no positive animals were identified in the Hot desert (BWh) regions. The GLMs revealed no significant associations between *Bartonella* prevalence and season, region, climate zone, or sex.

**Conclusions:**

This study demonstrated a high prevalence and diversity of zoonotic and canid-associated *Bartonella* species among wild canids in Israel, highlighting golden jackals as an important host species for *Bartonella* circulation in the region.

**Graphical Abstract:**

**Figure:**
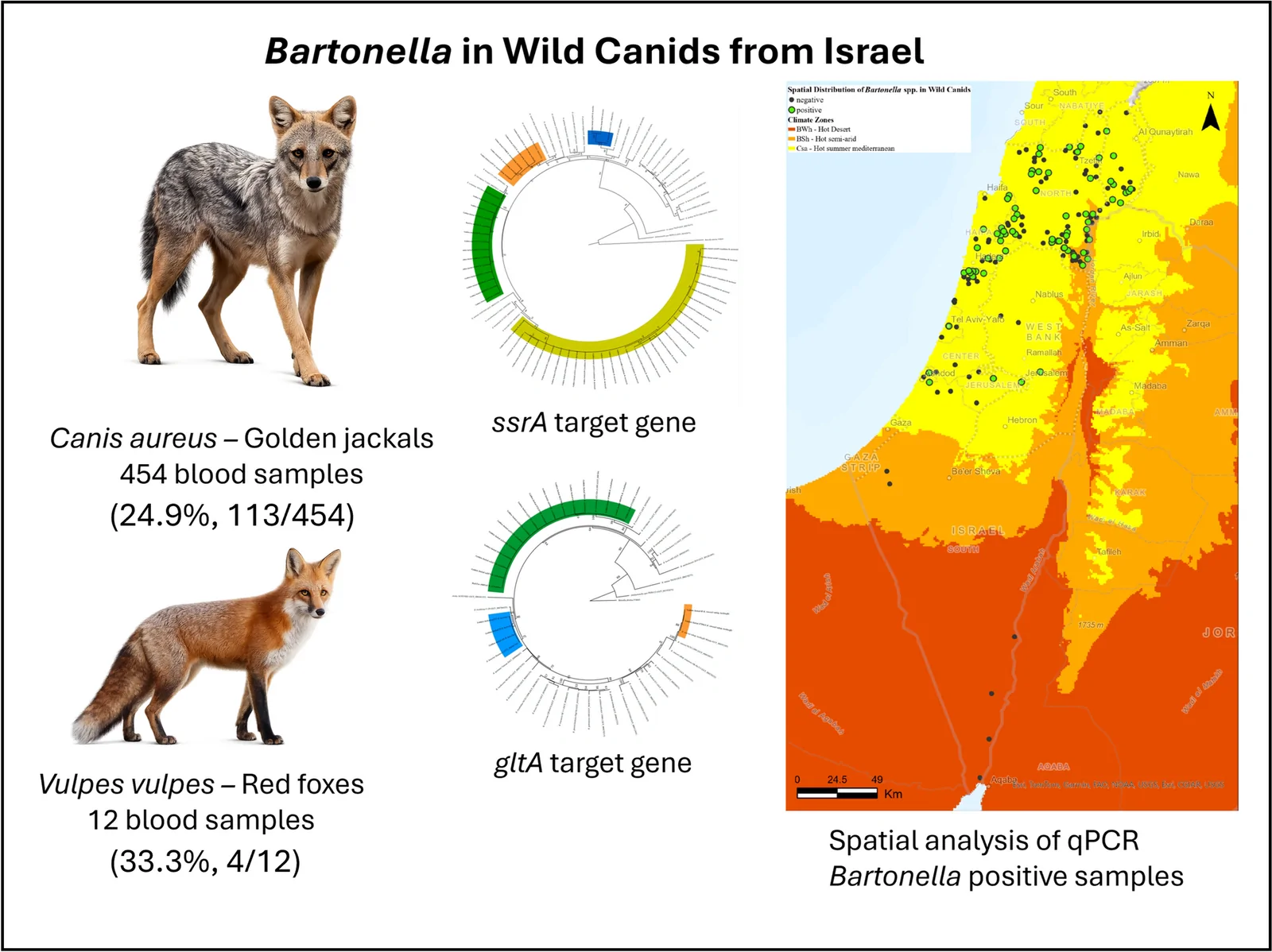

**Supplementary Information:**

The online version contains supplementary material available at https://doi.org/10.1186/s13071-026-07576-x.

## Background

*Bartonella* species are fastidious Gram-negative bacteria transmitted primarily by blood-feeding arthropods, with a wide diversity of mammalian reservoir hosts worldwide [1]. More than 50 *Bartonella* species have been described to date. Host specificity and phylogenetic associations with particular mammalian groups, including rodents, carnivores, ruminants, and bats, have been described [2–8]. Ecological overlap among wildlife, domestic animals, humans, and arthropod vectors may facilitate spillover events and the emergence of zoonotic transmission cycles.

Wild carnivores are potential reservoirs and sentinels for vector-borne pathogens, particularly in ecosystems undergoing environmental and anthropogenic changes [4, 9, 10]. Among carnivores, wild canids are highly adaptable species capable of occupying wild, peri-urban, and urban environments, increasing opportunities for interaction with domestic animals and shared ectoparasites [11, 12]. According to Kosoy and Goodrich (2019), two zoonotic *Bartonella* species have been identified in wild canids from the genera *Canis* and *Vulpes*, namely *Bartonella rochalimae* and *Bartonella vinsonii* subsp. *berkhoffii*, whereas ‘*Candidatus* Bartonella merieuxii’ has so far only been detected in animals of the genus *Canis*. Previous studies in the Mediterranean Basin and Middle East have demonstrated the circulation of *Bartonella* spp. among golden jackals (*Canis aureus*), red foxes (*Vulpes vulpes*), and domestic dogs, suggesting that wild canids may play an important role in maintaining and disseminating of these bacteria in nature [13–16].

Wildlife populations in Israel are exposed to environmental pressures, including urbanization, agricultural expansion, habitat fragmentation, and growing interactions with humans and domestic animals. These factors may influence vector abundance, host movement, and pathogen transmission dynamics [17, 18]. Previous studies conducted in Israel detected *Bartonella* DNA in wild canids, particularly in golden jackals and red foxes, indicating the circulation of zoonotic and canid-associated lineages in the region [13]. However, information regarding the eco-epidemiology, climatic associations, and genetic diversity of *Bartonella* spp. circulating among wild canids in Israel has remained limited, mainly due to restricted access to wildlife samples and the scarcity of long-term cross-sectional studies.

Therefore, the present study aimed to investigate the prevalence, phylogenetic diversity, and spatial distribution of *Bartonella* spp. among wild canids sampled across multiple ecological regions in Israel between 2018 and 2025. Additionally, we evaluated epidemiological and environmental variables associated with *Bartonella* detection, using a One Health framework integrating wildlife ecology, molecular epidemiology, and spatial analysis.

## Methods

### Sampling

Blood samples from wild canids were collected between 2018 and 2025 through the Israel Wildlife Disease Surveillance Program (IWDS) by the Israel Nature and Parks Authority (INPA) during predator and rabies control activities, from 158 sampling sites distributed across six geographical regions in Israel, including the North, Golan Heights, Center, Jerusalem, South, and West Bank regions. Three wild canid species were included in the study: golden jackals (*Canis aureus*), gray wolves (*Canis lupus*), and red foxes (*Vulpes vulpes*). For each sampled animal, epidemiological and ecological variables were recorded, including species, sex, season of collection (autumn, winter, spring and summer), geographical region, and climate zone. Climate zone classification was assigned according to the Köppen–Geiger climate classification system and categorized into three major climatic zones identified among the sampling sites. Geographic coordinates of each sampled animal were also recorded for spatial analyses and kernel density mapping. Blood samples were collected during routine wildlife surveillance and management activities and stored under appropriate conditions until molecular analysis was carried out.

### *Bartonella* molecular detection and phylogenetic analyses

DNA was extracted from canid blood samples using a commercial extraction kit (QIAamp DNA Blood Mini Kit, Qiagen®) according to the manufacturer’s instructions. The DNA samples underwent screening using real-time qPCR targeting *Bartonella* specific intergenic transcribed spacer (ITS) and the citrate synthase (*gltA*) loci, as previously described [19]. qPCR-positive samples underwent further conventional PCR by targeting the housekeeping genes/loci of *Bartonella* including gene fragments of the *gltA* and the transfer-messenger RNA (*ssrA*). DNA of the rodent-associated *Bartonella krasnovii* was used as a positive control [20]. The real-time qPCR assays were performed for all the DNA samples in 20 μl reaction volumes containing 2 μl of DNA, 0.6 μl of 50 μM solution of Syto9 (Invitrogen®), 0.5 μl of 10 mM of each primer, 10 μl Maxima Master Mix (Thermo Fisher Scientific®) and 6.4 μl ultrapure PCR water. The conventional PCR assay was performed in PCR-ready High Specificity mix (Syntezza Bioscience, Israel) in 25 μl reaction volumes containing 3 μl of DNA, 1 μl of 10 mM of each primer and 20 μl of ultrapure PCR water. Positive DNA amplicons were sequenced at the Center for Genomic Analyses at the Hebrew University (Jerusalem, Israel) using the BigDye Terminator cycle from Applied Biosystems ABI3700 DNA Analyzer.

The nucleotide sequences and their consensus were elucidated by a contig analysis run in Geneious®11.1.5 [21]. The comparisons with sequences deposited in Genbank were made using the basic local alignment search tool (BLAST®, National Center for Biotechnology Information, NCBI). Co-infection was considered when sequencing analyses showed different *Bartonella* species either from PCR amplicons generated from the same genetic locus or from different loci (e.g., ITS and *gltA*, or *gltA* and *ssrA*) within the same sample. The sequences of *gltA* and *srrA* for *Bartonella* spp. in this study were aligned using MEGA (Version 11) with species described in  previous studies [22, 23]. Phylogenetic trees were reconstructed using the maximum-likelihood method implemented in IQ-TREE v2 [24]. Model selection was conducted with ModelFinder integrated into IQ-TREE using the Bayesian Information Criterion (BIC) [25]. For both the *gltA* and *ssrA* alignments, the best-fit evolutionary model selected was HKY + F + I + G, and Ultrafast bootstrap analysis was used to evaluate branch support.

### Spatial distribution of *Bartonella*

To evaluate the distribution of *Bartonella* positive samples across geographic coordinates, positive detections were overlaid onto climate zone classifications. Climate data were obtained from a raster layer representing Köppen–Geiger climate zones, including Hot desert (BWh), Hot Semi-arid (BSh), and Hot Summer Mediterranean (Csa) regions. Spatial overlay analysis was performed in ArcGIS Pro (Version 3.3.0) to assign each sample to its corresponding climate category. The distribution of positive samples across climate zones was assessed descriptively. The resulting maps integrated point occurrence data with climatic regions to visualize the ecological context of pathogen distribution. This approach provides insight into potential associations between climate conditions and the spatial occurrence of positive cases, although it does not account for differences in sampling effort or host density across regions.

The spatial distribution of *Bartonella* positive wild canids was assessed using Kernel Density Estimation (KDE) also using ArcGIS Pro (Version 3.3.0). Positive samples were represented as point features based on their geographic coordinates (X, Y). Kernel density surfaces were generated to visualize the spatial intensity of positive detections across the study area. No population field was specified, and all points were equally weighted. The output raster values represent densities (points per unit area), enabling identification of areas with higher concentrations of positive samples. Density surfaces were classified using equal interval methods for visualization. The resulting maps reflect the relative spatial concentration of detected positive samples and do not represent true prevalence or infection risk, as patterns may be influenced by underlying sampling effort.

### Statistical analysis

Statistical analyses were performed in R version 4.5.2 (R Core Team, Vienna, Austria) using RStudio (Posit Software, Boston, MA, USA). The prevalence of *Bartonella* spp. according to species, sex, season, region, and climate zones were calculated as the number of positive samples divided by the total number of samples examined. Descriptive statistics, prevalence estimates, and 95% confidence intervals (CI) were calculated using the full dataset. Confidence intervals for prevalence estimates were calculated using the Wilson score method implemented in the *binom* package, and statistical significance was defined as *p* < 0.05 [26].

Associations between *Bartonella* infection status and explanatory variables were assessed using generalized linear models (GLMs) with a binomial error distribution and logit link function [27]. Season, region, climate zone, year, and sex were evaluated as potential predictors of *Bartonella* positivity. The variable year was retained in the analyses, because comparison of nested models with and without year indicated improved model fit. Global effects of explanatory variables were assessed using likelihood-ratio tests adjusted for the remaining variables in the model, implemented through the *Anova* function in the *car* package [28]. Pairwise comparisons among variable levels were performed using estimated marginal means with Tukey adjustment for multiple testing in the *emmeans* package [29].

Two separate models were fitted: (a) one model included season, region, climate zone, and year as explanatory variables for *Bartonella* prevalence; (b) the second model evaluated the association between sex and *Bartonella* prevalence independently, as sex information was unavailable for a substantial proportion of samples. Statistical significance was defined as *p* < 0.05. Both models were restricted to golden jackals as sample size for the red foxes and gray wolves was substantially low. Inclusion of these species in the models could have introduced additional source of variation and reduce the robustness of the analyses.

## Results

A total of 473 wild canids were included in the study, comprising 454 golden jackals, 12 red foxes, and 7 wolves. Overall, *Bartonella* DNA was detected in 117/473 animals (24.7%). Golden jackals showed a high prevalence of 24.9% (113/454; CI 20.9–28.9) and the red foxes showed a prevalence of 33.3% (4/12; CI 6.7–60.0). No positive wolves were detected (0/7; CI: 0–41.0) (Table 1). Seasonal variation in *Bartonella* prevalence among golden jackals ranged from 22.7% (37/163; CI 16.3–29.1) in winter to 30.4% (17/56; CI 18.3–42.5) in summer. Autumn and spring prevalence values were 26.4% (39/148; CI 19.3–33.5) and 23.0% (20/87; CI 14.1–31.9), respectively. Positive red foxes were detected in autumn and winter seasons only, although limited sample size should be noted (Table 1). Based on Israel’s geographical regions, most animals were sampled in northern Israel, where 25.2% (82/325; CI 20.5–29.9) of golden jackals were positive. The highest prevalence in golden jackals was observed in the Golan Heights region (32.5%; 13/40; CI 18.0–47.0), whereas the lowest prevalence was detected in southern Israel (7.7%; 1/13; CI 0–22.2). In Jerusalem, the two sampled red foxes were found positive (Table 1). According to climatic zones, most positive animals (golden jackals and red foxes) originated from Csa (Hot Summer Mediterranean), where golden jackals presented a prevalence of 25.9% (97/375; CI 21.5–30.3). In BSh (Hot Semi-arid), prevalence among golden jackals was 22.6% (14/62; CI 12.2–33.0), whereas no positive golden jackals were detected in BWh (Hot desert) (0/6) (Table 1). Sex analysis showed a slightly higher prevalence among female golden jackals (27.3%; 54/198; CI 21.1–33.5) compared to males (22.0%; 48/218; CI 16.5–27.5). Positive red foxes were detected only among males (40.0%; 2/5; CI 0–82.9) (Table 1).

**Table 1:** Prevalence of *Bartonella* spp. among wild canids according to species, season, geographic region, climate zone, and sex in Israel. Canine species: Golden jackal (*C. aureus*), Gray wolf (*C. lupus*), and Red fox (*V. vulpes*)

Variable	Category	Species	Total (n)	Positive (n)	Prevalence % (95% CI)**	GLM global p value***
Overall						
		*C. aureus*	454	113	24.9 (20.9–28.9)	
		*C. lupus*	7	0	0 (0–41.0)	
		*V. vulpes*	12	4	33.3 (6.7–60.0)	
Season*						
	Autumn	*C. aureus*	148	39	26.4 (19.3–33.5)	0.61
		*C. lupus*	3	0	0 (0–70.8)	
		*V. vulpes*	3	2	66.7 (13.3–100)	
	Winter	*C. aureus*	163	37	22.7 (16.3–29.1)	
		*C. lupus*	0	0	NA	
		*V. vulpes*	4	1	25.0 (0–67.4)	
	Spring	*C. aureus*	87	20	23.0 (14.1–31.9)	
		*C. lupus*	1	0	0 (0–95.0)	
		*V. vulpes*	3	0	0 (0–70.8)	
	Summer	*C. aureus*	56	17	30.4 (18.3–42.5)	
		*C. lupus*	3	0	0 (0–70.8)	
		*V. vulpes*	2	0	0 (0–84.2)	
Region†						
	Center	*C. aureus*	58	14	24.1 (13.1–35.1)	0.74
		*C. lupus*	0	0	NA	
		*V. vulpes*	0	0	NA	
	Golan	*C. aureus*	40	13	32.5 (18.0–47.0)	
		*C. lupus*	0	0	NA	
		*V. vulpes*	0	0	NA	
	Jerusalem	*C. aureus*	6	1	16.7 (0–46.5)	
		*C. lupus*	2	0	0 (0–84.2)	
		*V. vulpes*	2	2	100 (15.8–100)	
	North	*C. aureus*	325	82	25.2 (20.5–29.9)	
		*C. lupus*	3	0	0 (0–70.8)	
		*V. vulpes*	4	2	50.0 (1.0–99.0)	
	South	*C. aureus*	13	1	7.7 (0–22.2)	
		*C. lupus*	1	0	0 (0–95.0)	
		*V. vulpes*	3	0	0 (0–70.8)	
	West Bank	*C. aureus*	1	0	0 (0–95.0)	
		*C. lupus*	0	0	NA	
		*V. vulpes*	1	0	0 (0–95.0)	
Climate zone‡						
	Csa—hot summer	*C. lupus*	375	97	25.9 (21.5–30.3)	0.4
		*C. lupus*	4	0	0 (0–60.2)	
		*V. vulpes*	6	3	50.0 (10.0–90.0)	
	BSh—hot semi-arid	*C. aureus*	62	14	22.6 (12.2–33.0)	
		*C. lupus*	1	0	0 (0–95.0)	
		*V. vulpes*	1	1	100 (5.0–100)	
	BWh—hot desert	*C. aureus*	6	0	0 (0–45.9)	
		*C. lupus*	1	0	0 (0–95.0)	
		*V. vulpes*	3	0	0 (0–70.8)	
Sex§						
	Female	*C. aureus*	198	54	27.3 (21.1–33.5)	0.25
		*C. lupus*	1	0	0 (0–95.0)	
		*V. vulpes*	2	0	0 (0–84.2)	
	Male	*C. aureus*	218	48	22.0 (16.5–27.5)	
		*C. lupus*	4	0	0 (0–60.2)	
		*V. vulpes*	5	2	40.0 (0–82.9)	

^*^Season data were unavailable for 3 golden jackals

^†^Geographic region data were unavailable for 11 golden jackals and 2 red foxes

^‡^Climate zone classification was unavailable for 11 golden jackals and 2 red foxes

^§^Sex information was unavailable for 38 golden jackals, 2 wolves, and 5 red foxes

^**^Prevalence values are presented as percentages with 95% confidence intervals calculated using the Wilson score method. NA = not applicable due to absence of sampled individuals in the category

^***^Global p values correspond to likelihood-ratio tests for overall effect of each variable in a multivariable binomial generalized linear model (GLM), adjusted for the other variables in the model

Generalized linear models revealed no significant associations between *Bartonella* prevalence and season, region, climate zone, or sex for golden jackals. Pairwise comparisons among levels of these variables showed no significant differences (Supplementary data, S1). The only significant pairwise comparison was observed between samples of golden jackals collected in 2020 and 2025 (*p* = 0.04; Supplementary data, S1). The overall effect of year was not associated with consistent differences in prevalence across all sampling years. The difference between 2020 and 2025 may indicate temporal variation in *Bartonella* prevalence; however, differences in sample composition and sampling effort among years may have influenced this result, with lower prevalence (1/36) in 2020 than in 2025 (30/98).

‘*Candidatus* Bartonella merieuxii’ was the most prevalent *Bartonella* species detected in wild canids from Israel, identified in 53/454 (11.7%; CI 9.0–15.0) golden jackals (*Canis aureus*) and 3/12 (25%; CI 8.9–53.2) red foxes (*Vulpes vulpes*). *Bartonella rochalimae* was detected in 33/454 (7.2%; CI 5.2–10.0) golden jackals and 1/12 (8.3%; CI 1.5–35.4) red foxes. *Bartonella henselae* and *Bartonella vinsonii* subsp. *berkhoffii* were detected exclusively in golden jackals, with prevalence of 3.9% (18/454; CI 2.5–6.2) and 2.6% (12/454; CI 1.5–4.6), respectively. No detections of these species were observed in  the red foxes (Table 2). Additionally, co-infections of *Bartonella* species were found in the golden jackals (12/454, 2.6%). The most frequent co-infection was the simultaneous detection of ‘*Candidatus* B. merieuxii’ and *Bartonella henselae* (*n* = 4), followed by ‘*Candidatus* B. merieuxii’ with *B. rochalimae* (*n* = 3) and ‘*Candidatus* B. merieuxii’ with *B. vinsonii* subsp. *berkhoffii* (*n* = 3). Two golden jackals were co-infected with *B. rochalimae* and *B. vinsonii* subsp. *berkhoffii*. No co-infections were detected in the red foxes.

**Table 2:** Distribution and prevalence of *Bartonella* species detected in wild canids from Israel

*Bartonella* species	Golden jackal (*Canis aureus*)	Prevalence % (95% CI)*	Red fox (*Vulpes vulpes*)	Prevalence % (95% CI)*
‘*Candidatus* Bartonella merieuxii’	53/454	11.7 (9.0–15.0)	3/12	25.0 (8.9–53.2)
*Bartonella rochalimae*	33/454	7.2 (5.2–10.0)	1/12	8.3 (1.5–35.4)
*Bartonella henselae*	18/454	3.9 (2.5–6.2)	0/12	0
*Bartonella vinsonii* subsp. *berkhoffii*	12/454	2.6 (1.5–4.6)	0/12	0

^*^Prevalence values are presented as percentages with 95% confidence intervals calculated using the Wilson score method

**Fig. 1:**
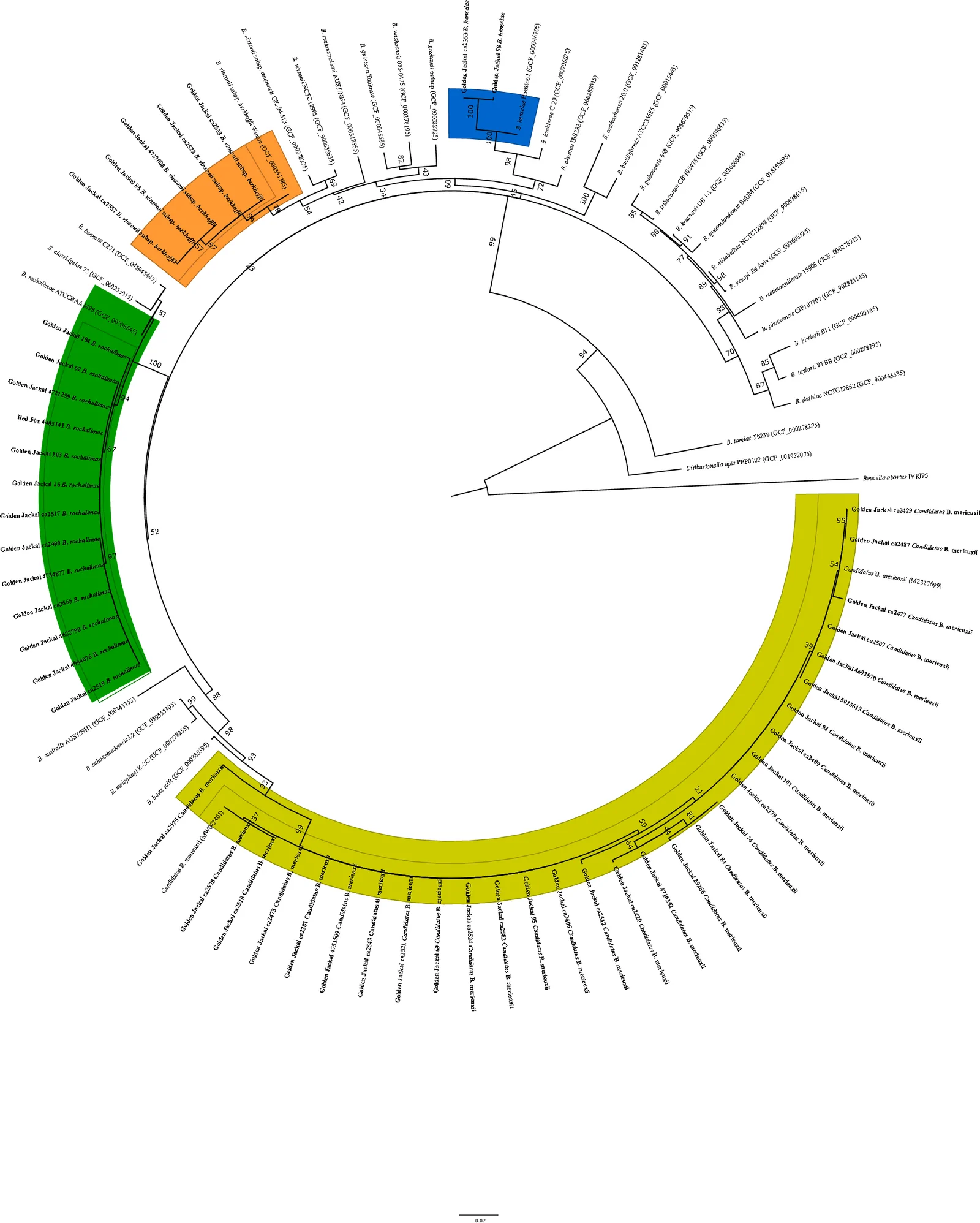
Phylogenetic relationships among *Bartonella* spp. inferred from partial *ssrA* gene sequences obtained from wild canids in Israel and Genbank available reference strains. The tree highlights the diversity of zoonotic and canid-associated *Bartonella* lineages circulating in wildlife reservoirs. Colored clades represent: green: *B. rochalimae* cluster; yellow: *Candidatus B. merieuxii* cluster; orange: *B. vinsonii berkhoffii* cluster; blue: *B. henselae*-like lineage

Phylogenetic analysis based on partial *ssrA* gene sequences demonstrated the circulation of zoonotic and canid-associated *Bartonella* species among wild canids in Israel (Fig. 1). The obtained sequences clustered into four major lineages corresponding with *Bartonella rochalimae*, ‘*Candidatus* Bartonella merieuxii*’*, *Bartonella vinsonii* subsp. *berkhoffii*, and *Bartonella henselae*-like. The sequences clustered with previously described reference strains available in GenBank.

**Fig. 2:**
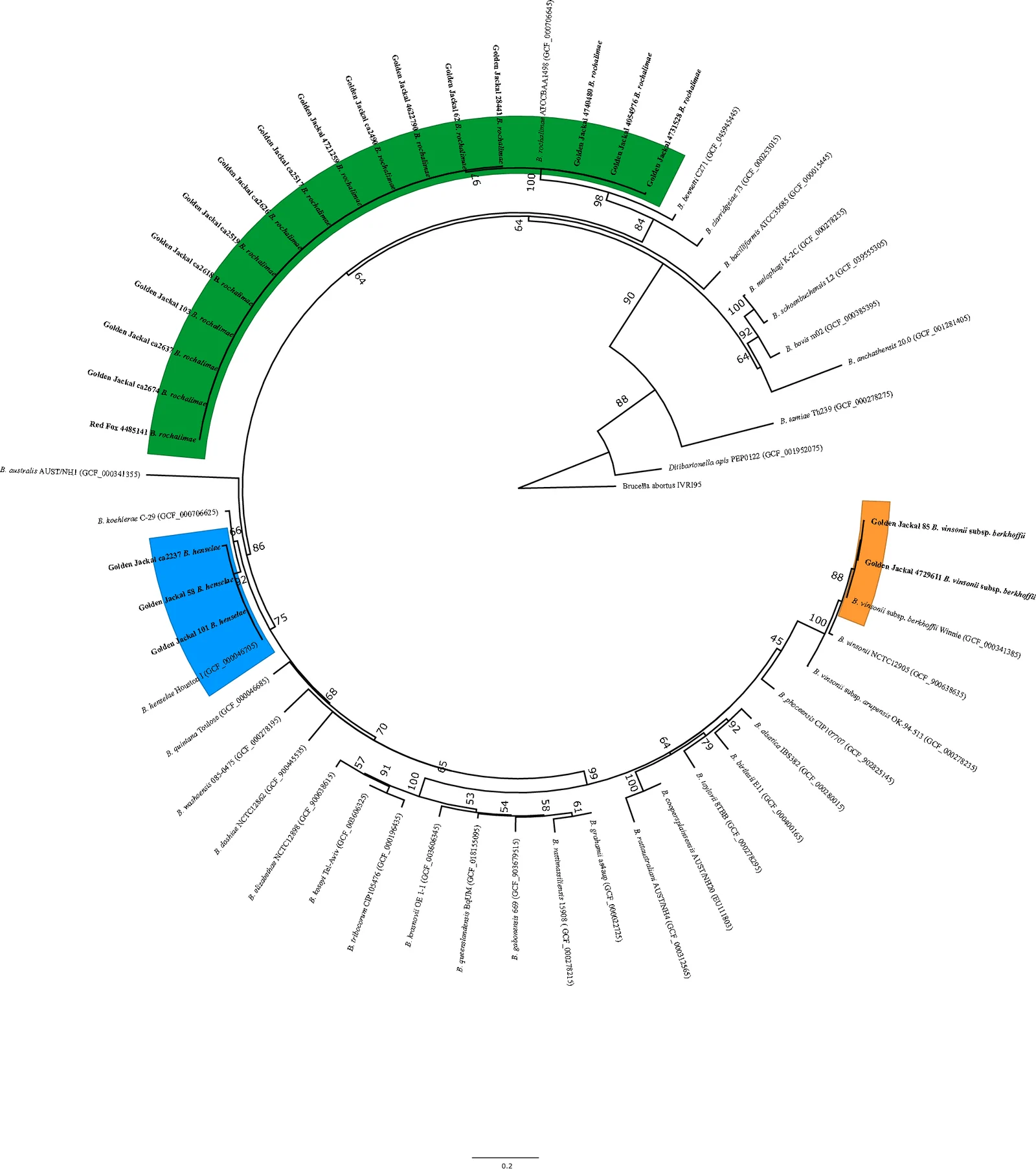
Phylogenetic relationships among *Bartonella* spp. based on partial *gltA* gene sequences obtained from wild canids in Israel and Genbank available reference strains. The tree highlights the diversity of zoonotic and canid-associated *Bartonella* lineages circulating in wildlife reservoirs. Colored clades represent: green clade: *B. rochalimae* cluster; blue clade: *B. henselae* cluster; orange clade: *B. vinsonii berkhoffii* cluster

Phylogenetic reconstruction based on partial *gltA* gene sequences showed a similar clustering pattern and further confirmed the genetic diversity of *Bartonella* species circulating among wild canids in Israel (Fig. 2). The *gltA*-based tree identified three main clades corresponding to *B. rochalimae*, *B. henselae* and *B. vinsonii* subsp. *berkhoffii*. The predominance of *B. rochalimae*-related sequences corroborated the findings obtained with the *ssrA* marker. The detection of *B. henselae* and *B. vinsonii* subsp. *berkhoffii* further demonstrated the coexistence of multiple pathogenic *Bartonella* lineages in wild canid populations across different ecological regions of the country (Fig. 2).

**Fig. 3:**
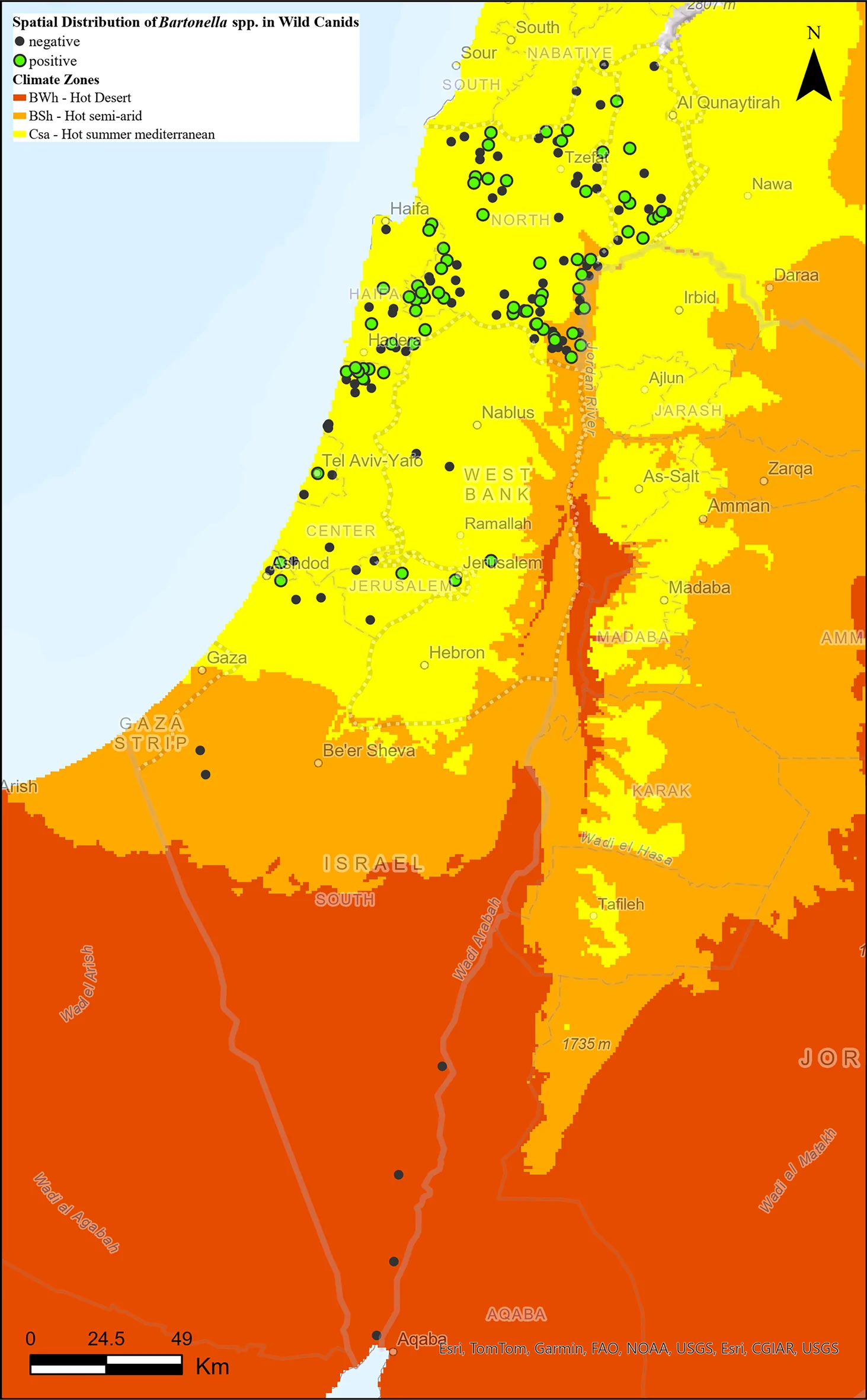
Spatial distribution of *Bartonella* spp. detected in wild canids sampled across Israel, Judea, and Samaria regions. Sampling locations are classified as positive (green circles) or negative (black circles) for *Bartonella* spp. detection. The background colors represent Köppen–Geiger climate zones, including BWh (Hot desert), BSh (Hot semi-arid), and Csa (Hot Summer Mediterranean). Positive samples were predominantly distributed in Mediterranean and semi-arid regions of northern and central Israel

Spatial analyses showed that positive samples were only distributed in the Hot Summer (Csa) and Hot Semi-arid (BSh) regions of northern and central Israel, whereas no positive samples were identified in the desert (BWh) areas (Fig. 3).

**Fig. 4:**
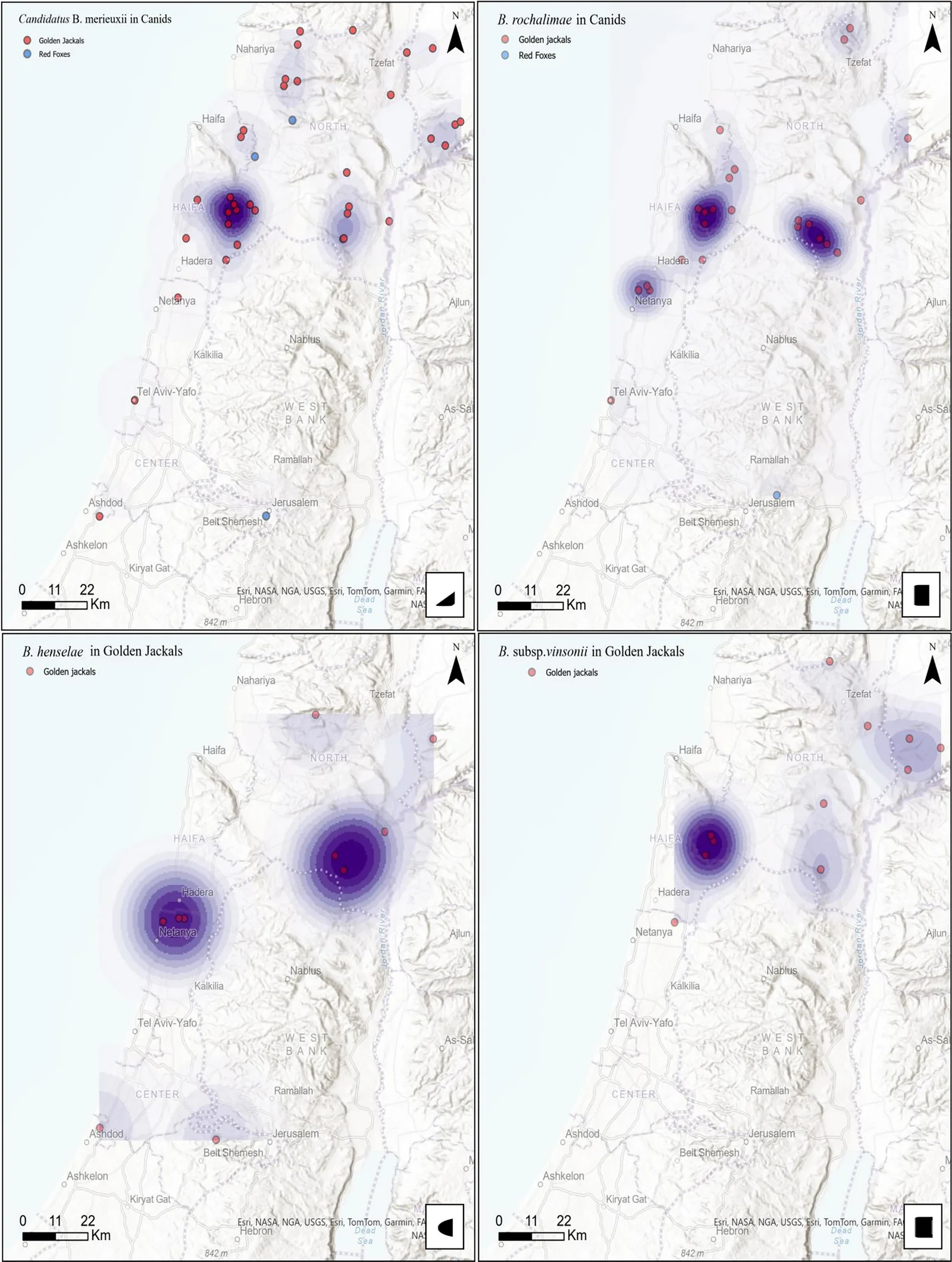
Kernel density maps showing the spatial distribution of *Bartonella* species detected in wild canids across Israel. (4**A**) *Candidatus* Bartonella merieuxii; (4**B**) *Bartonella rochalimae*; (4**C**) *Bartonella henselae*; and (4**D**) *Bartonella vinsonii* subsp. *berkhoffii*. Red circles represent positive golden jackals (*Canis aureus*), while blue circles represent positive red foxes (*Vulpes vulpes*) when applicable. The purple gradient indicates kernel density intensity based on the concentration of positive records, with darker areas corresponding to higher densities of infected animals. Major hotspots were identified in the Menashe Heights, Beit-Shean Valley, the Ha’Sharon Plain coastal region, northern Israel, indicating focal circulation patterns of different Bartonella species among wild canid populations across the country

Kernel density analyses (KDE) of the four *Bartonella* spp. showed focal geographic hotspots at Northern Israel, in Menashe Heights, Beit-Shean Valley and Ha’Sharon Plain, respectively (Fig. 4).

## Discussion

This study presents a high prevalence and diversity of *Bartonella* spp. among wild canids in Israel. It highlights the role of these animals as important reservoirs of zoonotic and canid-associated *Bartonella* lineages in this region. Four *Bartonella* spp. were detected in the canines, including *'Candidatus* Bartonella merieuxii*'*, *Bartonella rochalimae*, *Bartonella henselae*, and *Bartonella vinsonii* subsp. *berkhoffii*. Among the sampled canids, the golden jackal (*Canis aureus*) showed an overall prevalence of 24.9%, while red foxes (*Vulpes vulpes*) showed 33.3%, although the red fox sample size was limited (*n* = 12). These findings indicate that wild canids, especially golden jackals, in Israel harbor multiple phylogenetically distinct *Bartonella* species, including taxa with zoonotic relevance.

The most frequently detected species was '*Candidatus* Bartonella merieuxii', identified in both jackals and red foxes (Table 2, Figs. 1 and 4). *‘Candidatus* B. merieuxii’ was originally described in domestic dogs and golden jackals from Iraq, where it was detected in approximately 37% of dogs and 12% of jackals, suggesting that canids may represent major reservoir hosts for this species [15]. Previous studies have already identified this candidate species in dogs from the Mediterranean and Middle Eastern regions, supporting its broad regional circulation among canids [13, 16]. The high frequency observed in the present study suggests that *'Candidatus* B. merieuxii' may represent one of the dominant *Bartonella* species circulating among wild canids in Israel and neighboring countries.

The detection of *Bartonella rochalimae* in 8.3% of the canids indicates the circulation of zoonotic canid-associated *Bartonella* species in wild canids from Israel (Table 2). *Bartonella rochalimae* has previously been associated with carnivores, such as foxes, coyotes, raccoons and domestic dogs, and human infections have also been reported [14, 30]. In Israel, Marciano et al. [13] previously identified *B. rochalimae*-like organisms in golden jackals and red foxes, demonstrating that this species has likely been circulating in the local wildlife populations. The phylogenetic clustering observed in both the *ssrA* and *gltA* analyses in the present study further confirms the close relationship between the detected sequences and previously characterized *B. rochalimae* strains (Fig. 1 and 2).

The detection of *B. henselae* is a noteworthy finding of this study in golden jackals. Although *B. henselae* is associated with domestic cats and cat fleas, spillover from the latter to non-feline hosts has been described [4, 31–33]. The presence of *B. henselae* DNA in wild canids may reflect ecological overlap among wild and domestic canines and felines, as well as arthropod vectors, particularly in peri-urban or human-modified environments. Golden jackals are highly adaptable carnivores that frequently exploit urban and agricultural landscapes, increasing the likelihood of contact with domestic animals and flea vectors. The detection of *B. henselae*, a feline-associated zoonotic agent, in wild canids reinforces the importance of a "One Health" perspective when evaluating *Bartonella* circulation dynamics in regions where wildlife, domestic animals and humans coexist closely.

The prevalence of *Bartonella* spp. observed in the present study appears higher than that reported in earlier wildlife studies conducted in Israel. Marciano et al. [13] reported *Bartonella* positivity in approximately 13% of golden jackals, whereas the present study identified positivity in 24.9% of sampled jackals suggesting an increase in the prevalence. Several factors may contribute to this increase, including increased interactions among wildlife, domestic animals and arthropod vectors which may influence transmission dynamics over time. Additionally, environmental changes, such as urbanization, habitat fragmentation, increased pollution, and human interventions that affect the natural balance, may have contributed to this increase in prevalence. It should be noted that substantially larger sample size, broader geographic areas, and greater seasonal coverage were included in this study compared to the previous one. Nevertheless, because samples in the present study were collected over multiple years and across heterogeneous regions, these differences should not be considered as evidence of a direct temporal increase in infection rates.

The performed spatial analyses showed that the occurrence of *Bartonella* spp. in wild canids was predominantly at northern Israel (Figs. 3 and 4); however, the climate zones related to *Bartonella* prevalence were not statistically significant (Table 1). This may reflect ecological conditions favorable for vector survival and host density, particularly in Mediterranean environments characterized by higher humidity and vegetation cover compared with arid desert ecosystems, consistent with the previous reports demonstrating the influence of climatic conditions on the distribution of arthropod-borne zoonotic pathogens in the Eastern Mediterranean region [34]. However, the KDE analyses presented here reflect concentrations of positive detections rather than true prevalence or transmission risk, since they do not account for differences in sampling effort, host abundance, or vector density across regions.

Despite the molecular detection of '*Candidatus* B. merieuxii', the authors attempted multiple times to isolate this bacterium from blood samples; however, bacterial isolation was unsuccessful. One possible explanation is that long-term storage conditions and sample preservation may not have been optimal for maintaining bacterial viability [19]. *Bartonella* species are known to be fastidious and difficult to culture, particularly when samples undergo prolonged storage, repeated freeze–thaw cycles or suboptimal transport conditions. Similar challenges have been reported in previous studies involving wildlife-associated *Bartonella* spp. [19]. Additionally, amplification of the *gltA* locus was not successful for '*Candidatus* B. merieuxii' in the present study, despite previous reports that successfully managed to so [13, 15]. This may reflect low bacterial load, DNA degradation, primer mismatch, or sequence divergence affecting amplification efficiency. The inability to amplify all housekeeping loci highlights the challenges of molecular characterization of wildlife-associated *Bartonella* species and suggests that future studies may benefit from broader genomic approaches or optimized primer sets targeting divergent lineages.

The dataset of this study was dominated by golden jackals, whereas wolves and foxes were represented by relatively small sample size, limiting interspecies comparisons. It has to be noted that golden jackals dominate the wild canid population in Israel whereas red foxes and gray wolves are present in much lower numbers. Furthermore, the cross-sectional nature of the study does not allow direct inference regarding transmission dynamics or seasonal incidence. Finally, because the spatial analyses did not normalize for sampling effort or host density, identified hotspots should be interpreted as areas of concentrated positive detections rather than definitive estimates of infection risk (Fig. 4).

## Conclusions

The present study expands the understanding of *Bartonella* diversity and eco-epidemiology among wild canids in the Middle East. The detection of multiple zoonotic and canid-associated species, including *B. henselae*, *B. rochalimae* and *Bartonella vinsonii* subsp. *berkhoffii*, highlights the importance of wild canids as potential sentinels and reservoirs within regional transmission networks. These findings reinforce the importance for integrated "One Health" surveillance strategies combining wildlife, domestic animals, arthropod vectors, and human populations to understand better the transmission dynamics and public health implications of *Bartonella* circulation in Israel and neighboring countries.

## Supplementary Information

Supplementary material 1.

Supplementary material 2.

## Data Availability

No datasets were generated or analyzed during the current study.
