# Supplementary material for: Wild canids as reservoirs of zoonotic Bartonella species in Israel
Source: Parasit Vectors. 2026 Jul 15;19:441. doi: 10.1186/s13071-026-07576-x (PMC13651138; doi:10.1186/s13071-026-07576-x)
Supplement: Supplementary file 1 [file 13071_2026_7576_MOESM2_ESM.docx]

**Supplementary Data**

**Table S1**. Estimated marginal means and pairwise comparisons for explanatory factors from binomial generalized linear models (GLMs) of *Bartonella* positivity.

| Variable | Contrast | Odds ratio | 95% CI | Adjusted p-value** |
| --- | --- | --- | --- | --- |
| Season |  |  |  |  |
|  | autumn / spring | 1.12 | 0.4–3.14 | 0.99 |
|  | autumn / summer | 0.86 | 0.34–2.23 | 0.98 |
|  | autumn / winter | 1.39 | 0.61–3.16 | 0.74 |
|  | spring / summer | 0.77 | 0.25–2.4 | 0.94 |
|  | spring / winter | 1.24 | 0.51–3.03 | 0.93 |
|  | summer / winter | 1.61 | 0.6–4.32 | 0.61 |
| Region |  |  |  |  |
|  | Center / Golan | 1.11 | 0.29–4.28 | 1 |
|  | Center / North | 1.21 | 0.45–3.28 | 0.96 |
|  | Center / South | 3.11 | 0.16–59.95 | 0.76 |
|  | Golan / North | 1.09 | 0.41–2.95 | 1 |
|  | Golan / South | 2.81 | 0.15–52.71 | 0.8 |
|  | North / South | 2.57 | 0.15–43.2 | 0.83 |
| Climate zone* |  |  |  |  |
|  | BSh / Csa | 0.75 | 0.38–1.47 | 0.4 |
| Year |  |  |  |  |
|  | year2019 / year2020 | 11.12 | 0.58–214.63 | 0.17 |
|  | year2019 / year2023 | 1.17 | 0.35–3.97 | 1 |
|  | year2019 / year2024 | 0.92 | 0.33–2.52 | 1 |
|  | year2019 / year2025 | 0.6 | 0.17–2.08 | 0.79 |
|  | year2020 / year2023 | 0.11 | 0.01–2 | 0.23 |
|  | year2020 / year2024 | 0.08 | 0–1.45 | 0.12 |
|  | year2020 / year2025 | 0.05 | 0–0.94 | **0.04** |
|  | year2023 / year2024 | 0.78 | 0.29–2.1 | 0.96 |
|  | year2023 / year2025 | 0.51 | 0.17–1.58 | 0.48 |
|  | year2024 / year2025 | 0.65 | 0.27–1.59 | 0.68 |
| Sex |  |  |  |  |
|  | female / male | 1.31 | 0.83–2.08 | 0.25 |

Pairwise comparisons were based on estimated marginal means from binomial GLMs. Estimates are shown as odds ratios with asymptotic 95% confidence intervals; p-values were adjusted using Tukey’s method.

*Csa – Hot Summer, BSh – Hot Semi-arid
